# Mecp2 Mediates Experience-Dependent Transcriptional Upregulation of Ryanodine Receptor Type-3

**DOI:** 10.3389/fnmol.2017.00188

**Published:** 2017-06-13

**Authors:** Rodrigo F. Torres, Cecilia Hidalgo, Bredford Kerr

**Affiliations:** ^1^Laboratory of Biology, Centro de Estudios CientíficosValdivia, Chile; ^2^Biomedical Neuroscience Institute, Centro de Estudios Moleculares de la Célula, Department of Neuroscience and Physiology and Biophysics Program, Instituto de Ciencias Biomédicas, Facultad de Medicina, Universidad de ChileSantiago, Chile

**Keywords:** MeCP2, ryanodine receptor, epigenetics, experience-dependent plasticity, transcriptional regulation

## Abstract

Mecp2 is a DNA methylation reader that plays a critical role in experience-dependent plasticity. Increasing evidence supports a role for epigenetic modifications in activity-induced gene expression. Hence, candidate genes related to such phenomena are of great interest. Ryanodine receptors are intracellular calcium channels that contribute to hippocampal synaptic plasticity, dendritic spine remodeling, and participate in learning and memory processes. Here we exposed mice to the enriched environment (EE) paradigm, which through increased stimulation induces experience dependent-plasticity, to explore a role for methyl-cytosines, and Mecp2 in directing Ryanodine receptor 3 (*Ryr3*) transcriptional activity. EE induced a hippocampal-specific increase in the methylation of discrete cytosines located at a *Ryr3* isoform promoter; chromatin immunoprecipitation experiments revealed that EE increased Mecp2 binding to this *Ryr3* isoform promoter. Interestingly, the experimental paradigm induced robust *Ryr3* upregulation, accompanied by *miR132*-dependent suppression of *p250GAP*, a pathway driving synaptogenesis. In contrast to WT mice, *Mecp2-null* mice showed diminished levels of *Ryr3* and displayed impaired EE-induced *Ryr3* upregulation, compromising *miR132* dependent suppression of *p250GAP* and experience-dependent structural plasticity. Based on these results, we propose that Mecp2 acts as a transcriptional activator of *Ryr3*, contributing to experience-dependent plasticity.

## Introduction

The former picture of immutable epigenetic modifications has given rise to a landscape that is dynamic in its nature and range of responses (Guo et al., 2011; Irier et al., 2014). This dynamic landscape includes modifications in post-mitotic neurons, such as post-translational modifications of histones and methylation/hydroxymethylation of cytosines (Cortes-Mendoza et al., 2013; Guo et al., 2014; Lopez-Atalaya and Barco, 2014). Cytosine methylation is essential for regulating gene expression (Bird, 2002; Schubeler, 2015). Interestingly, cytosine methylations is sensitive to neuronal activity (Guo et al., 2011) and increasing evidence supports the involvement of such modification in synaptic plasticity and learning and memory processes (Miller et al., 2010; Day and Sweatt, 2011; Morris and Monteggia, 2014; Tognini et al., 2015).

MECP2 is a methylation reader with a dual role on gene expression; its function in the nervous system is highlighted by the phenotype observed in patients and mouse models of Rett syndrome (RTT), a devastating neurodevelopment disorder caused by mutations in the X-linked *MECP2* gene (Chahrour and Zoghbi, 2007). Although the participation of MECP2 in directing gene expression is widely accepted and extends over several cellular processes (Chahrour et al., 2008; Chen et al., 2015), the mechanisms that link this methylated cytosine binding protein to neuronal plasticity processes remain poorly understood. In previous reports it was proposed that altered experience-dependent plasticity contributes to Rett syndrome pathogenesis (Zoghbi, 2003; Noutel et al., 2011; Della and Pizzorusso, 2014). Therefore, elucidating MECP2-target genes that contribute to activity-dependent neuronal remodeling is important to understand the basis of this complex neurodevelopmental disorder.

The concentration of intracellular free calcium is highly regulated during activity-induced synaptic plasticity and is essential for activity-induced gene expression (Bading, 2013; Paula-Lima et al., 2014). Ryanodine receptors (RyR) are intracellular calcium release channels, which through calcium-induced calcium release contribute to hippocampal synaptic plasticity (Wang et al., 1996; Grigoryan et al., 2012) and dendritic spine remodeling (Adasme et al., 2011; Lesiak et al., 2014). RyR channels contribute to activity-dependent dendritic spine formation by modulating the Rac1-PAK actin remodeling pathway through *miR132*-dependent suppression of the Rho-family GTPase Activating Protein p250GAP (Wayman et al., 2008; Lesiak et al., 2014). Two of the three mammalian RyR isoforms, RyR2 and RyR3, are involved in learning and memory (Galeotti et al., 2008; Adasme et al., 2011), and transcriptional activity of *Ryr2* and *Ryr3* is increased in rat hippocampus after spatial memory training in the Morris water maze (Adasme et al., 2011). These findings prompt interest in unraveling the mechanisms underlying transcriptional regulation of these calcium channels and their contribution to experience-dependent plasticity. Particularly, *Ryr3* deletion caused reduced AMPA-mediated synaptic responses and impaired hippocampal long term potentiation (Shimuta et al., 2001), whereas *Ryr3* knockdown impaired hippocampal-dependent memory (Galeotti et al., 2008), suggesting that this calcium channel is essential to hippocampal function.

Altered expression of the *Ryr3* gene was detected when comparing wild type (WT) and *Mecp2-null* mice (Ben-Shachar et al., 2009; Zhao et al., 2013). Moreover, methylation of the *Ryr3* gene promoter is sensitive to neuronal activity (Guo et al., 2011). We explored the role of Mecp2 together with cytosine methylation in directing transcriptional activity of the *Ryr3* calcium channel in mice exposed to an enriched-environment (EE), a widely used paradigm known to induce experience-dependent plasticity (Nithianantharajah and Hannan, 2006; Baroncelli et al., 2010). We found that EE induces modifications in the methylation of discrete cytosines located at the *Ryr3* isoform-specific promoter. These modifications are hippocampus-specific and related to transcriptional upregulation of the *Ryr3* gene. Concordantly, we found that Mecp2 binds to the proximal promoter of the *Ryr3* gene and that EE increased this interaction in WT mice. Moreover, *Mecp2*-null mice showed diminished *Ryr3* mRNA levels when compared to WT mice housed in standard conditions (SC). We further showed that Mecp2 absence impairs *Ryr3* upregulation, compromising *miR132*-induced *p250GAP* downregulation and experience-dependent structural plasticity elicited by EE. Altogether, our results indicate that methylation together with Mecp2 activate *Ryr3* transcription, contributing to experience-dependent structural plasticity.

## Methods

### Animals

In order to reduce the number of mice and minimize the variation as consequence of genetic background, all experiments of this study were performed with mice on the 129/SvJ background. Colony founders for *Mecp2*-null mice (Guy et al., 2001) were obtained from The Jackson Laboratory stock #003890. Heterozygous *Mecp2*-null female mice were inbred and only *Mecp2*-null male mice and their WT male littermates were used. Mice were kept under 12–12 h light-dark cycles. Food and water were provided *ad libitum*. Unless stated otherwise, mice were euthanized at 8 weeks of age. Experiments were approved by the Centro de Estudios Cientificos Animal Care and Use Committee. The mouse facility of the Centro de Estudios Cientificos is accredited by the Association for the Assessment and Accreditation of Laboratory Animal Care International (AAALAC).

### Enriched environment

We used a previously described EE paradigm (Kerr et al., 2010) with minor modifications. After weaning (p21), mice were placed in either enriched or standard environments until euthanasia at 8 weeks of age. The EE condition used larger cages (795 cm^2^ for SC and 1590 cm^2^ for EE) and larger animal groups (6 mice in SC and 10 mice in EE) compared to standard housing conditions; EE cages included pet toys of several shapes and colors and a voluntary running wheel. To increase novelty, toys were changed on a daily basis. Mice were weekly controlled for body weight and presented no statistically significant differences compared to SC housed mice.

### RNA isolation and real time PCR

RNA was isolated and reverse transcribed as previously described (Torres-Andrade et al., 2014). Briefly, brains were dissected and samples were homogenized in Trizol according to manufacturer's instructions. RNA was precipitated and treated with one unit of DNase I (Life Technologies). Five micrograms of total RNA were reverse transcribed using random primers and ImProm II kit (Promega). cDNA was quantified by qPCR using Kapa SYBR Quantimix (Kapa). The qPCR analysis was performed in triplicates from one reverse transcribed product using the Rotor Gene 6000 (Corbett). Values were analyzed following the 2^−ΔΔCt^ method using cyclophilin-A (*Cyc1*) and β2-microglobulin (*B2m*) as normalization controls, using the following primer pairs: *Ryr3*, F: TGGTGTCGGTGATGATCTGT, R: TGCACAGGTTGTCCATTGAT (1); Cyc1, F: GGCAATGCTGGACCAAACACAA, R: GTAAAATGCCCGCAAGTCAAAAG; B2m, F: GCTATCCAGAAAACCCCTCAA, R: CATGTCTCGATCCCAGTAGACGGT (Torres-Andrade et al., 2014). Experiments were repeated at least twice.

### Micro RNA132 relative quantification

For *miR132* quantification, the mirVana miRNA isolation kit was used (Life technologies) followed by individual TaqMan Small RNA assays (Life technologies) as previously described (Myklebust et al., 2011). The assays used were *miR132* (461735_mat) and snoRNA234 (001234). 15 ng of DNAse-treated RNA were reverse transcribed using the TaqMan MicroRNA Reverse Transcription Kit (P/N 4366596) according to the manufacturer instructions. Real time was performed using the TaqMan Universal PCR Master Mix II, No UNG (P/N 4440040) and TaqMan Assay according to instructions. Quantification was performed according to the 2^−ΔΔCt^ method, using snoRNA234 as an endogenous small-RNA normalization control. *Cyc1* from the same samples was also evaluated to corroborate sample quality.

### DNA extraction and bisulphite sequencing

Brain samples were homogenized and digested by Proteinase-K at 55°C. Following treatment with RNase, DNA was extracted by phenol/chloroform/isoamylic acid 25:24:21 (Invitrogen) according to the manufacturer's instructions. After precipitation, DNA quality was corroborated by 260/280 absorbance ratio >1.7 and gel analysis. Targeted bisulphite next generation sequencing was performed by Zymo Research Corporation (Irvine, CA) using the following primers sets RyR3_1 F: TTTAGATGTTTGTTTGTGTAAAGTTTGTGG, R: CAACCCTACCCAAAAACATACCTAAATAAT RyR3_2 F: TAGGAAATTTGATTTTATTGTGTAGTGTTT R: AACCTCTTCCCCCAAAAATATAAAC and RyR3_3 F: TAATTAAGATTGAAAGAGTAGATTTGTTTAGAT R: AACCACCTAAAAATAAACTTAATTATCAAAATAA. Sequence reads were identified using standard Illumina base-calling software and the methylation level of each sampled cytosine was estimated as the number of reads reporting a C, divided by the total number of reads reporting a C or T. Sequenced samples showed a mean total read number of 97,460, an average CpG coverage of 7861X and a bisulphite conversion rate of 99%. The region of interest (chr2:113.029.482-113.031.331) was obtained from USCS genome browser (http://genome.ucsc.edu/) considering 1000 base pairs upstream and 800 base pairs downstream from the transcription start site from the *Ryr3* isoform identified by the code uc0081pg.1 (Genomic sequence: chr2: 112.631.382-113.030.331). The region of interest comprises 9 CpGs in this 1,800 bp region surrounding the first exon of this *Ryr3* isoform. Other *Ryr3* isoforms might not be directed by the region analyzed in our study. Samples from hippocampus (HPC), cortex (CTX), and cerebellum (Cb) were obtained from each mouse.

### Chromatin immunoprecipitation

Chromatin immunoprecipitation was performed from whole hippocampal samples, using the MAGnify Chromatin Immunoprecipitation System (Life Technologies) according to manufacturer's instructions. Briefly, samples were homogenized and cross-linked before proceeding to 15 min of sonication in cycles of 30 s. Agarose gel analysis showed chromatin fragments enriched in the 100–300 base-pair range. Three microgram of anti-Mecp2 antibody (ab2828, Abcam) were used for each immunoprecipitation and IgG was used as a control. Mecp2 antibody specificity was corroborated by using samples from *Mecp2*-null mice. The primer pair used to assay Mecp2 binding to the *Ryr3* promoter was F: TGCATAGAGCAAACGCAGGT and R: AGAGCATGCCTAAGTGGTCG. Values were analyzed by the 2^−ΔΔCt^ method relative to SC, and the H19 locus was used as an immunoprecipitation control for Mecp2 (Drewell et al., 2002; Zhou et al., 2006) using the following primers F: GGGGTTCACCTGTTTTGCAC and R: GGCTTTTGTGCTTTCTGGCA. Three biological replicates were used.

### Dendritic spine density

Golgi-Cox impregnation was performed on brain slices using the Rapid Golgi staining kit (FD Neurotechnologies) following the manufacturer's instructions. Secondary or tertiary dendrites from the *striatum radiatum* of the CA1 region were photographed from coronal sections of the hippocampus using a MSHOT camera (Digital Microscope Camera MD-90) mounted over an Olympus CX31 microscope. Images had 3,488 × 2,616 pixels and were taken at 100x magnification. The images were processed as previously described to obtain the digital skeleton of the dendrites (Orlowski and Bjarkam, 2012). Dendritic spines were counted using the imageJ software and the skeleton analysis function. Dendritic spine density was estimated from 3 animals (8 week-old) per condition in 20–30 dendrites per mouse.

### Morris water maze

To evaluate spatial memory, the Morris water maze was used. This assay was performed as previously described with few modifications (Adasme et al., 2011). Briefly, the pool (120 cm diameter) was filled with water (22–24°C) until a deep of 50 cm was reached. The water was made opaque by the use of non-toxic white paint and spatial cues were placed surrounding the pool at a height of 1 m. The test was performed for 4 consecutive days and four 60 s trials were completed each day. The platform (10 × 15 cm) was hidden during all sessions. During the first trial on the first day, mice were placed in the pool for 1 min; if the platform was not found, mice were taken to the platform and given time (25 s) to observe the spatial cues. All sessions were video-recorded to register escape latency. Two days after training, *Mecp2-null* mice reared in EE were evaluated using a visible platform. Animals were euthanized 6 h after the last session and the whole hippocampus was recovered. *Mecp2-null* mice reared in SC exhibited significant impaired motor coordination and hence could not be tested in the Morris water maze.

## Results

**Figure 1:**
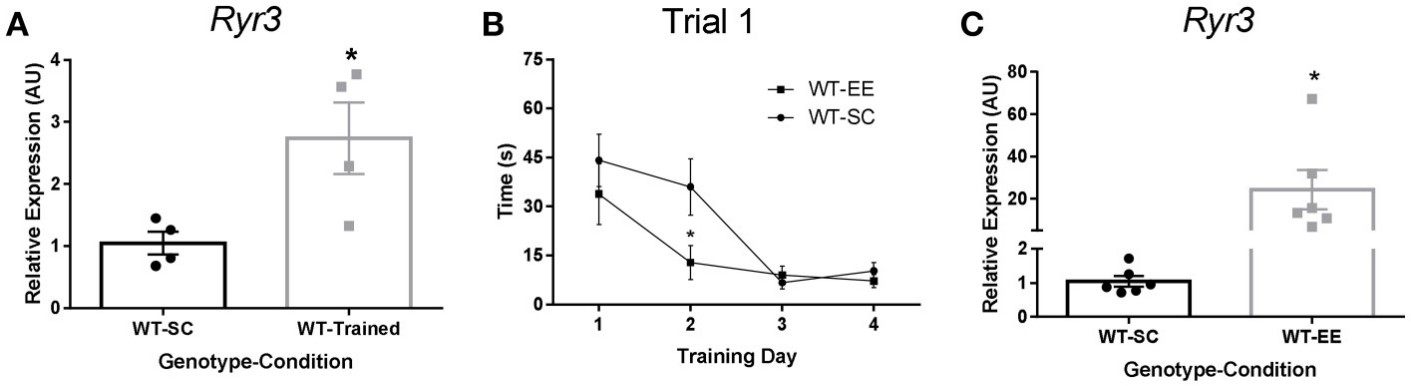
Spatial training and enriched environment increase hippocampal *Ryr3* mRNA. **(A)** Hippocampal *Ryr3* mRNA quantification for WT trained mice relative to SC housed mice, *t*-test, ^*^p < 0.05 (*n* = 4). **(B)** Escape latency registered for the first trial of each training day for WT mice housed in SC (*n* = 8) or in EE (*n* = 6). Statistical significance was analyzed by two-way ANOVA with Sidak's multiple comparison test, ^*^p < 0.05; Environmental condition, *p* < 0.05; Training day, *p* < 0.0001; interaction, *p* = 0.1502. **(C)** Hippocampal *Ryr3* mRNA quantification for animals reared in EE relative to SC (*t*-test, ^*^p < 0.05) (*n* = 4). Data are presented as mean ± SEM.

We first evaluated whether spatial learning promoted *Ryr3* transcriptional upregulation in mice. We measured *Ryr3* mRNA levels in whole hippocampal extracts from mice trained to find a hidden platform in the Morris water maze. We observed that spatial learning induced an increase of *Ryr3* mRNA levels compared to WT mice that did not undergo water maze training (Figure 1A). In addition, WT mice reared under EE conditions displayed a reduction in escape latency compared to WT mice housed in SC (Figure 1B). These observations together suggest that EE facilitates spatial learning and validate mice as a model to study the regulation of *Ryr3* by an experience-dependent plasticity paradigm.

To determine if the EE paradigm also upregulated *Ryr3* transcriptional activity, we measured *Ryr3* mRNA levels by qPCR in whole hippocampal extracts from mice reared in either SC or EE. We found that WT mice reared in EE had *Ryr3* mRNA levels 20-fold higher than WT mice maintained in SC (Figure 1C). This result was confirmed by evaluating another two cohorts of mice (Supplementary Figure 1). These observations support the use of mice reared in EE to study transcriptional regulation of *Ryr3* and its contribution to experience-dependent plasticity.

**Figure 2:**
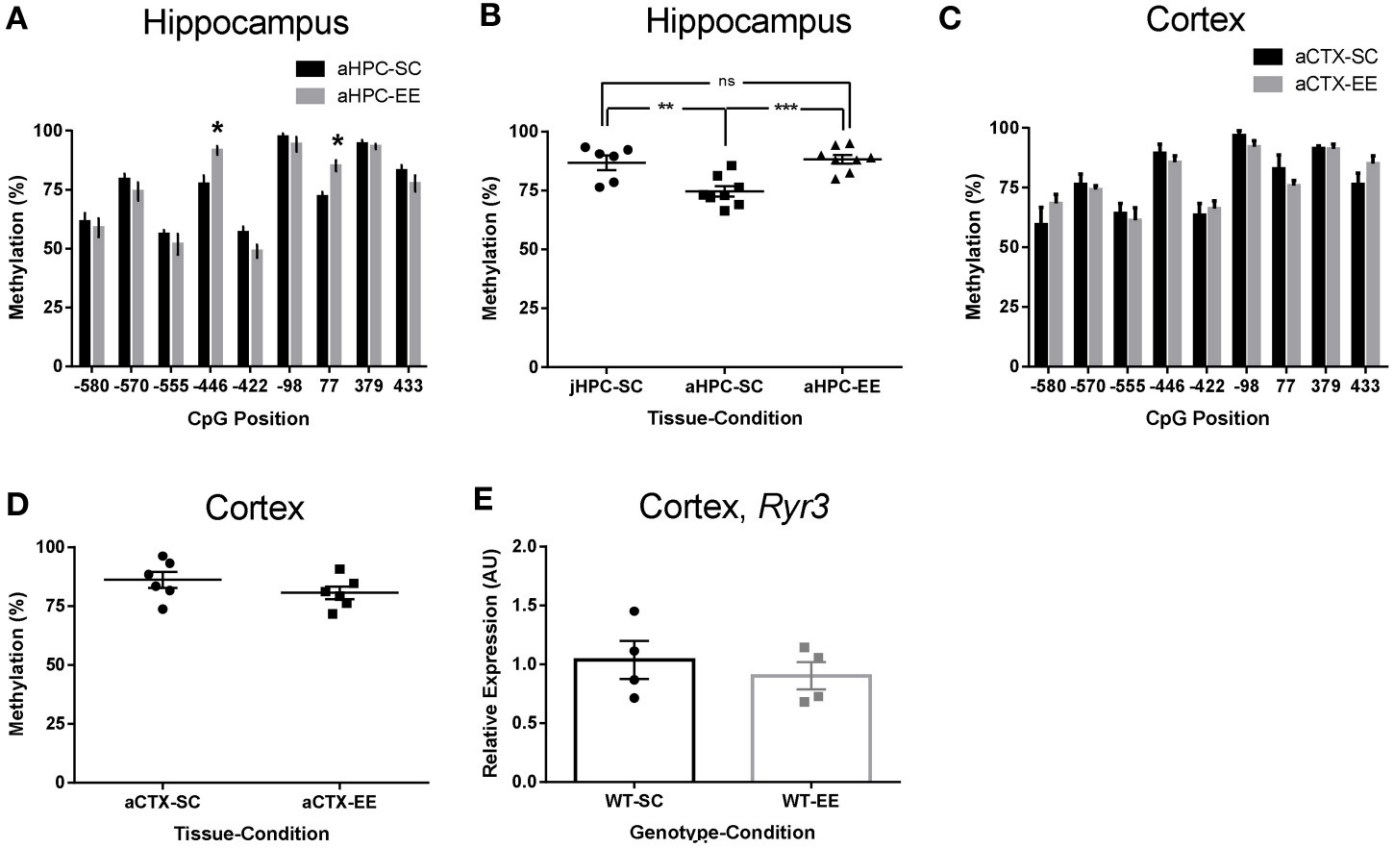
Enriched environment-induced increased in Ryr3 promoter methylation in the hippocampus. **(A)** Hippocampal (HPC) DNA methylation percentage profile for cytosines surrounding the *Ryr3* transcription start site for mice reared in standard conditions (SC, *n* = 4) or enriched environment (EE, *n* = 4). Positions relative to the transcription start site. ^*^Differences relative to SC, *t*-test, *p* < 0.05 for cytosine −446; *p* < 0.01 for cytosine 77. **(B)** Mean hippocampal methylation percentage for cytosines −446 and 77 in juvenile (j, P21, *n* = 3) or adult (a, 8 weeks. *n* = 4) animals reared in SC or EE; ANOVA with Tukey's multiple comparison test, ^**^p < 0.01, ^***^p < 0.001, ns, non-significant. **(C)** Cortical DNA methylation percentage profile for cytosines from mice reared in standard or enriched environment. Cytosine methylation were evaluated as shown in **(A)**. **(D)**, Methylation percentage for cytosines –446 and +77 estimated for cortex (CTX) samples from the animals used in **(A)**. **(E)**, Cortex *Ryr3* mRNA relative quantification for WT animals housed in SC and EE (*n* = 4). Data are presented as mean ± SEM.

To assess if *Ryr3* transcriptional upregulation was associated to changes in cytosine methylation, we isolated hippocampal DNA from 8 week-old WT mice and bisulfite-sequenced an 1,800 bp region located 1,000 bp upstream and 800 bp downstream of the transcription start site of a *Ryr3* isoform (uc0081pg.1). We found a high percentage of cytosine methylation within the analyzed *Ryr3* region (Figure 2A). Despite this fact, compared to methylation observed in WT mice reared in SC, mice reared under EE conditions displayed a significant increase in the methylation percentage of cytosines –446 (77 ± 3% for SC and 91 ± 1% for EE) and 77 (72 ± 2% for SC and 85 ± 3% for EE; Figure 2A).

In order to evaluate whether EE induces an increase in cytosine methylation or prevents a decrease in cytosine methylation, we next evaluated the mean methylation percentage of cytosines –446 and 77 in p21 juvenile mice, the age at which mice were weaned to either SC or EE cages. We found that in young mice the methylation percentage of cytosine –446 and 77 was similar to those of adult mice reared in EE (Figure 2B), and higher than those of adult mice housed in SC. Based on these results, we suggest that maturity decreases the methylation levels of cytosines –446 and 77 and that this decrease is prevented by the EE. Of note, the EE-induced methylation observed for cytosines –446 and +77 in the hippocampus from adult mice was not observed in the cortex (Figures 2C,D) or cerebellum (data not shown), suggesting that the observed increase of methylation induced by EE is hippocampus-specific. To gain further insight into the role of methylation in directing *Ryr3* transcriptional activity, we evaluated *Ryr3* expression in cortex of EE-reared mice and observed no differences in *Ryr3* mRNA levels compared to SC (Figure 2E). Similar results were found in cerebellum (data not shown). Therefore, we suggest that *Ryr3* transcriptional upregulation is associated to EE-induced methylation of the *Ryr3* gene promoter in mouse hippocampus.

**Figure 3:**
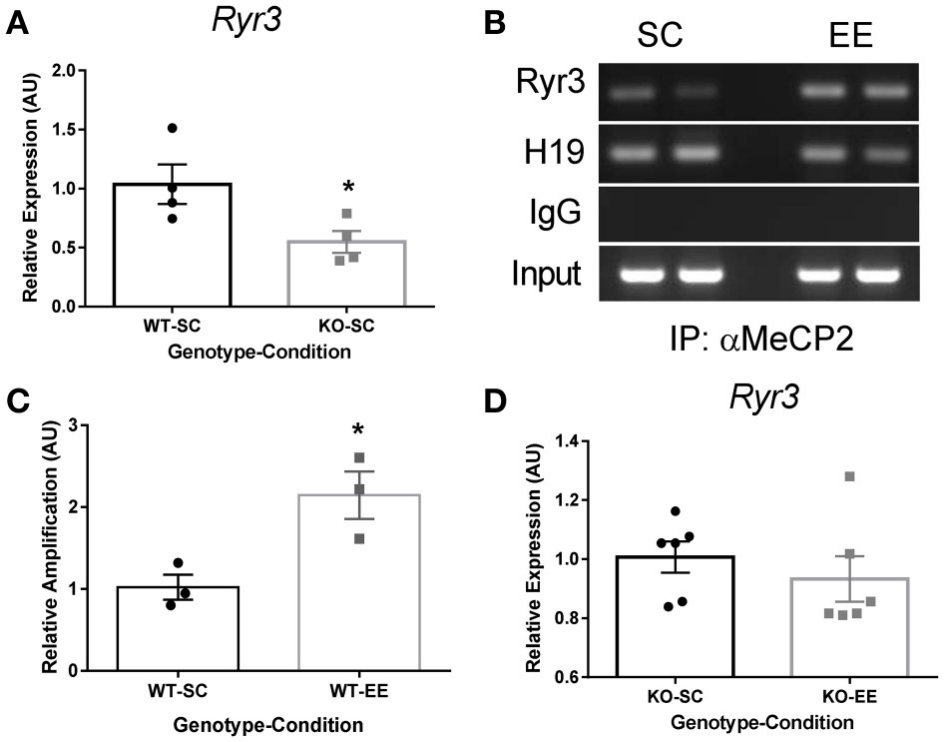
Mecp2 mediates EE-induced transcriptional upregulation of *Ryr3*. **(A)** Hippocampal *Ryr3* mRNA quantification for *Mecp2-null* mice housed in SC relative to WT (*t*-test, ^*^p < 0.05; *n* = 4) **(B)** Mecp2 ChIP analysis by PCR directed to the *Ryr3* gene promoter and the *H19* locus as a control, IgG: immunoglobulin. **(C)** Mecp2 immunoprecipitate, qPCR quantification directed to the *Ryr3* gene promoter relative to SC samples; *t*-test, ^*^p < 0.05 (*n* = 3) **(D)**, Hippocampal *Ryr3* mRNA quantification for *Mecp2-null* mice reared in EE relative to the SC (*n* = 6). Data are presented as mean ± SEM.

To decipher how an increase in methylation of discrete cytosines leads to changes in gene expression, we hypothesized that the methylated DNA reader Mecp2 participates in transcriptional regulation of the *Ryr3* gene. Consequently, we evaluated *Ryr3* mRNA levels in *Mecp2-null* mice and observed that these mice displayed significantly reduced levels compared to WT mice reared in SC (Figure 3A). These results support the involvement of Mecp2 in the transcriptional regulation of *Ryr3*. To further support this observation, we evaluated Mecp2 binding to the *Ryr3* isoform proximal promoter by chromatin immunoprecipitation (ChIP) in WT mice reared in either EE or SC. We observed direct interaction of Mecp2 with the proximal promoter of *Ryr3* in samples from WT mice reared in SC (Figure 3B). qPCR analysis revealed a 2-fold increase of the *Ryr3* promoter immunoprecipitate when chromatin was obtained from WT mice exposed to EE compared to mice reared in SC (Figure 3C). These results reveal that Mecp2 acts as a transcriptional activator of the *Ryr3* gene in experience-dependent plasticity. To further test the role of Mecp2 in transcriptional regulation of *Ryr3*, we evaluated *Ryr3* mRNA levels in EE-reared *Mecp2-null* mice. We observed that in the absence of Mecp2, EE did not elicit an increase in *Ryr3* mRNA levels (Figure 3D). These results emphasize the role of Mecp2 in the transcriptional upregulation of *Ryr3* in this experience-dependent plasticity paradigm.

**Figure 4:**
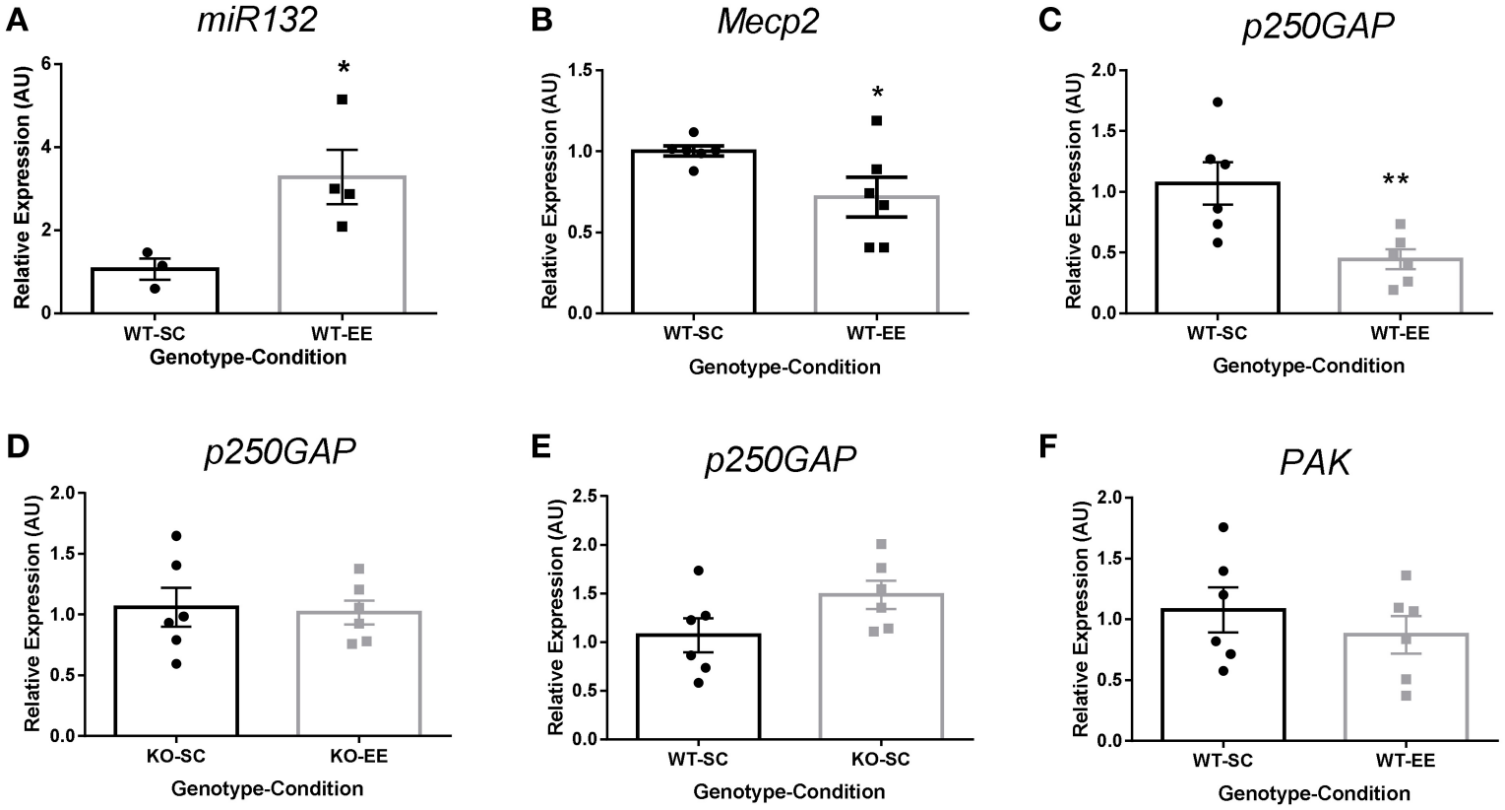
EE-induced *p250GAP* regulation is abolished in *Mecp2-null* mice: **(A)** Hippocampal *miR132* quantification for EE-reared (*n* = 4) WT mice relative to SC (*n* = 3) (*t*-test, ^*^p < 0.05). **(B)**, *Mecp2* mRNA quantification for WT mice reared in EE relative to SC (*t*-test, ^*^p < 0.05; *n* = 6). **(C)**, *p250GAP* mRNA quantification for WT mice reared in EE relative to SC (*t*-test, ^**^p < 0.01; *n* = 6). **(D)**, *p250GAP* mRNA quantification for *Mecp2-null* mice reared in EE relative to *Mecp2-null* mice reared in SC (*n* = 6). **(E)**, Relative *p250GAP* mRNA quantification for *Mecp2-null* mice reared in SC relative to WT mice reared in SC; *t*-test, ^**^p < 0.01 (*n* = 6). **(F)**, Relative *PAK* mRNA expression in WT mice reared in EE compared to WT reared in EE (*n* = 6). Data are presented as mean ± SEM.

*In vitro* experiments have shown that Ryr channels contribute to *miR132* upregulation-dependent suppression of p250GAP, contributing to dendritic spine formation by activating the Rac1-PAK actin remodeling signaling pathway (Lesiak et al., 2014). To determine if *Ryr3* upregulation is associated to *p250GAP* regulation in experience-dependent plasticity, we evaluated *miR132*-dependent *p250GAP* downregulation in WT mice reared in SC or EE. We observed that EE induced a 3-fold increase of *miR132* compared to SC housed WT mice (Figure 4A). We then evaluated the *miR132* targets *Mecp2* and *p250GAP*. We observed a significant reduction in *Mecp2* mRNA (Figure 4B) and a reduction of 60% in *p250GAP* mRNA levels (Figure 4C), suggesting that the increase of *miR132* elicited by EE is functional and effective in reducing its *Mecp2* and *p250GAP* mRNA targets. Moreover, *Mecp2-null* mice did not show *p250GAP* downregulation when housed in EE (Figure 4D), suggesting that Mecp2 contributes to downregulate *p250GAP* in experience-dependent plasticity. To determine if Mecp2 contributes directly to *p250GAP* regulation, we evaluated *p250GAP* mRNA levels in *Mecp2-null* mice. We observed no differences in *p250GAP* mRNA levels when compared to WT mice reared in SC (Figure 4E), suggesting that *p250GAP* is not a direct transcriptional target of Mecp2. PAK is a downstream effector of Rac1 in the actin remodeling pathway. We observed no changes in PAK mRNA levels (Figure 4F), suggesting that EE-elicited *Mecp2* and *p250GAP* downregulation are specific to *miR132* targets.

**Figure 5:**
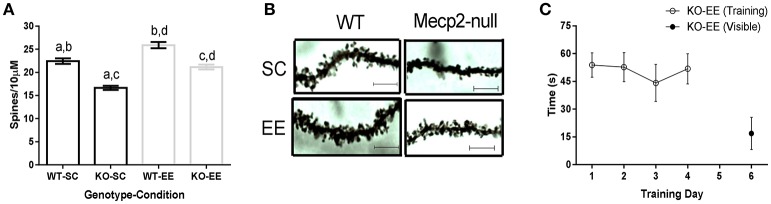
EE-induced experience-dependent structural plasticity is altered in *Mecp2-null* mice: **(A)**, Hippocampal dendritic spine density estimation for WT and *Mecp2-null* mice reared in SC or EE; ANOVA with Tukey's multiple comparison showed significant differences when comparing a–d (*n* = 3, 20–30 dendrites per mouse). A, *p* < 0.05; b, *p* < 0.05; c, *p* < 0.05; d, *p* < 0.05. **(B)**, Representative image of dendrites from WT and *Mecp2-null* mice reared in SC and EE (Bar = 5 μm). **(C)**, EE-reared *Mecp2-null* mice escape latency registered for the first trial of each training day (open circles) and when a visible platform was used on the sixth day (closed circle) (*n* = 6). Data are presented as mean ± SEM.

The *miR132* target *p250GAP* is a negative regulator of synaptogenesis. To evaluate if *p250GAP* downregulation is associated to dendritic spine remodeling in experience-dependent plasticity, we measured hippocampal dendritic spine density of pyramidal neurons in WT and *Mecp2-null* mice reared in SC or EE. WT animals showed EE-induced experience-dependent structural plasticity, revealed by increased dendritic spine density when compared to littermates housed in SC (Figure 5A). *Mecp2-null* mice had reduced dendritic spine levels (16.65 ± 0.44 dendritic spines per 10 μm) compared to WT mice housed in SC (22.43 ± 0.61 dendritic spines per 10 μm; Figure 5A). EE induced recovery of *Mecp2-null* mice dendritic spine density to a level comparable to WT-SC. However, the difference in dendritic spine density observed when comparing WT (25.90 ± 0.69 dendritic spines per 10 μm) and *Mecp2-null* mice (21.15 ± 0.50 dendritic spines per 10 μm) housed in EE suggests that experience-induced structural plasticity is compromised in *Mecp2-null* mice (Figures 5A,B).

To determine whether altered experience-dependent structural plasticity is functionally relevant, we evaluated EE-reared *Mecp2-null* mice in the Morris water maze. Unexpectedly, we observed that *Mecp2-null* mice housed in EE showed impaired spatial learning (Figure 5C). EE has been shown to improve motor function and coordination of *Mecp2-null* mice (Kerr et al., 2010). However, to corroborate that defective learning was not caused by swimming inability, we evaluated EE-reared *Mecp2-null* mice using a visible platform and observed that *Mecp2-null* mice reared in EE were able to swim and reach the platform (Figure 5C), suggesting that the contribution of Mecp2 to experience-dependent plasticity is crucial for spatial learning.

## Discussion

Seminal work in elucidating gene expression alterations caused by the lack of Mecp2 has shown either increased or decreased expression of target genes. *Ryr3* was among the genes with diminished expression in the cerebellum of *Mecp2*-null mice (Ben-Shachar et al., 2009; Zhao et al., 2013). Our results corroborate and extend this observation to the hippocampus and suggest that Mecp2 is required for the expression of basal *Ryr3* mRNA levels. Chromatin immunoprecipitation assays showed direct interaction of Mecp2 with the *Ryr3* isoform promoter used in this work. Other *Ryr3* isoforms might not be directed by the promoter region we focused in this work, thus, elucidating their transcriptional regulation remains an open question. Interestingly, EE increased Mecp2 interaction with the *Ryr3* isoform promoter in WT mice, supporting the involvement of Mecp2 in directing the transcriptional activity of the *Ryr3* isoform not only in basal conditions, but also in experience-dependent plasticity. Moreover, the expression of *Ryr2* mRNA was also increased by water maze training and by our experience-dependent plasticity paradigm; but interestingly, *Mecp2-null* mice showed unaltered *Ryr2* expression in relation to WT mice, suggesting that Mecp2 is not involved in directing *Ryr2* transcriptional activity (Supplementary Figure 2).

Previous work showed that electroconvulsive shock (ECS) increases *Ryr3* gene methylation (Guo et al., 2011). Our EE paradigm represents a natural and less invasive approach to increase neuronal activity and comprises an interesting model to study epigenetic modifications underlying gene expression regulation in experience-dependent plasticity. We observed a robust EE-induced increase in *Ryr3* mRNA that could be a direct consequence of increased transcriptional activity, alternative splicing or could also be accompanied by accumulation of mRNAs during the EE protocol. Further work must be done to account for these latter variables. Although the study by Guo et al. (2011) revealed increased methylation at the *Ryr3* gene, this was accompanied by a decreased expression of *Ryr3*. This discrepancy in the effect of increased methylation over *Ryr3* mRNA may be due to differences in the times at which samples were studied (4 h post-ECS vs. 5 weeks in the EE), or may arise from the differences in brain regions studied (dentate gyrus vs. whole hippocampus). It is also possible that different *Ryr3* isoform are directed in opposite directions by methylation. Despite these differences, it is interesting to note that *Ryr3* gene methylation increases either under an artificial or a natural paradigm, acting to increase neuronal activity.

Sparse CpGs are likely to be methylated (Rollins et al., 2006); however sparse CpG are also targets of stimulus-induced site-specific methylation modifications (Guo et al., 2011). Here we studied 9 CpGs located in a low CpG-density region and as expected, a high degree of methylation was observed. The next generation sequencing results were obtained by sequencing DNA extracted from total hippocampal tissue, which is comprised by several cellular types, some of them not expressing *Ryr3*. This heterogeneity is likely to result in an underestimation of the differential methylation levels. Notwithstanding, EE increased methylation of discrete cytosines located at the *Ryr3* proximal promoter of a specific *Ryr3* isoform. Brain plasticity undergoes an age-dependent decline that is ameliorated by EE (Baroncelli et al., 2010) and *Ryr3* is downregulated by aging (Schafer et al., 2015). Therefore, it was interesting to find that cytosines that showed an EE-induced increase in methylation showed similar increased levels of methylation in juvenile mice, suggesting that EE recovers or maintains methylation levels of these cytosines in adult mice. Remarkably, methylation modifications were only observed in the hippocampus. Other brain regions that did not show increased methylation, also failed to show the transcriptional response to the EE, highlighting the relation between CpG methylation and *Ryr3* transcriptional activity.

The Rho-family GTPase Activating Protein p250GAP is a negative regulator of synaptogenesis that modulates the Rac1-PAK actin remodeling signaling pathway (Wayman et al., 2008; Lesiak et al., 2014); it was suggested that RyR channels contribute to activity-induced synaptogenesis through *miR132*-dependent suppression of p250GAP (Lesiak et al., 2014). Interestingly, in WT mice EE-induced *Ryr3* upregulation is accompanied by increased *miR132* and diminished p250GAP, a recognized *miR132* target (Wayman et al., 2008). Therefore, it is likely that increased *miR132* drives the *p250GAP* downregulation observed in mice reared in the EE conditions. Moreover, the finding of unaltered levels of *p250GAP* mRNA displayed by *Mecp2-null* mice compared to WT mice housed in SC suggests that *p250GAP* is not subjected to Mecp2-direct transcriptional regulation. The absence of Mecp2 abolished transcriptional regulation of both, *Ryr3* and *p250GAP*, suggesting that Mecp2 contributes to experience dependent plasticity through *Ryr3*-direct and p250GAP-indirect regulation. Interestingly, it was shown that activation of Rho GTPases rescues neurobehavioral abnormalities displayed by Mecp2-308 male and female mice (De et al., 2012; De Filippis et al., 2015). Nevertheless, the mechanism by which the absence of Mecp2 impairs modulation of Rho GTPases is currently unknown, highlighting our observations about the contribution of Mecp2 to *p250GAP* regulation in experience-dependent plasticity. It is also of note that as a *miR132* target (Klein et al., 2007), *Mecp2* mRNA levels were reduced in the hippocampus of EE-reared mice, suggesting that environmental stimulation diminishes Mecp2 expression. Interestingly, similar observations have been reported for cultured cortical neurons exposed to stimuli inducing neuronal activation (Tropea et al., 2016). Our results extend this *in vitro* observation to a mouse model subjected to an environmental paradigm that increases neuronal activity. It has also been described that Mecp2 expression knockdown in primary hippocampal neurons results in increased expression of miR132 (Su et al., 2015), suggesting that there is a regulation interplay between Mecp2 and miR132. These observations together reveal a role of neuronal activity on *Mecp2* expression and a possible regulatory feedback for Mecp2-directed *Ryr3* transcriptional upregulation observed in EE.

EE is a paradigm widely used to induce experience-dependent plasticity (Baroncelli et al., 2010; Jung and Herms, 2014). Moreover, EE ameliorates several neurological diseases (Nithianantharajah and Hannan, 2006). *Mecp2-null* mice show major phenotypic improvement when housed in EE, suggesting that some key features of RTT can be bypassed by environmental stimulation (Kerr et al., 2010; Lonetti et al., 2010). However, our results show that *Mecp2*-null mice exposed to EE recovered dendritic spine density to a level only comparable to WT mice housed in SC, but not to WT mice reared in EE. Hence, we suggest as previously proposed (Lesiak et al., 2014), that *Ryr3* and *p250GAP* regulation contribute to activity-induced dendritic spine formation. Despite the major phenotypic improvement and recovered dendritic spine density, EE-housed *Mecp2-null* mice showed impaired spatial learning contrasting to the spatial learning facilitation elicited by EE in WT mice, highlighting the contribution of Mecp2 to experience-dependent plasticity and cognitive processes.

Recent work has shown that Mecp2 and specifically its MBD domain, is necessary for the maintenance of normal dendritic development (Chapleau et al., 2009; Zhao et al., 2015), suggesting that methylation modifications underlie the transcriptional changes that induce plasticity processes. The RyR3 calcium channel is an interesting candidate relating Mecp2 to experience-dependent plasticity. However, many other candidate genes remain to be studied. Altogether, our data propose a role for methylated cytosines together with Mecp2 in directing the basal transcriptional activity of a *Ryr3* isoform and its upregulation induced by EE, contributing to experience-dependent plasticity.

## Author contributions

RT, CH, and BK designed the research. RT performed the research. RT and BK analyzed the data. RT, CH, and BK wrote the article.

### Conflict of interest statement

The authors declare that the research was conducted in the absence of any commercial or financial relationships that could be construed as a potential conflict of interest.

## Supplementary material

Supplementary Figure 1 Relative *Ryr3* expression in WT mice. Mice from three cohorts were randomly assigned to SC or EE. Cohort 1 included 6 mice in EE and 6 mice in SC, cohort 2 included 4 mice in EE and 4 in SC and cohort 3 included 3 mice in EE and 3 mice in SC. Values represent mean ± SEM (*t*-test, ^***^*p* < 0.0001).

Supplementary Figure 2 Relative *Ryr2* expression in different conditions. (A) Hippocampal *Ryr2* mRNA quantification for WT mice reared in EE relative to SC housed mice; *t*-test ^***^*P* < 0.001 (*n* = 4). (B), Hippocampal *Ryr2* mRNA quantification for WT mice trained in the Morris maze relative to SC housed mice that were not trained; *t*-test ^*^*p* < 0.05) (*n* = 4). (C) Expression of *Ryr2* in *Mecp2-null* mice relative to WT mice reared in SC (*n* = 4).
