# Supplementary material for: Mecp2 Mediates Experience-Dependent Transcriptional Upregulation of Ryanodine Receptor Type-3
Source: Front Mol Neurosci. 2017 Jun 13;10:188. doi: 10.3389/fnmol.2017.00188 (PMC5468404; doi:10.3389/fnmol.2017.00188)
Supplement: Supplementary Figure 1 — Relative Ryr3 expression in WT mice. Mice from three cohorts were randomly assigned to SC or EE. Cohort 1 included 6 mice in EE and 6 mice in SC, cohort 2 included 4 mice in EE and 4 in SC and cohort 3 included 3 mice in EE and 3 mice in SC. Values represent mean ± SEM (t-test, ***p < 0.0001). [file Image1.pdf]

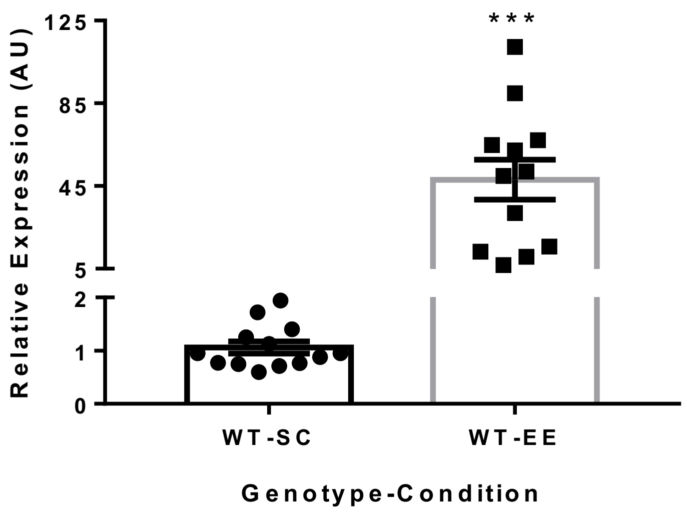

Supplemental Figure 1: Relative *Ryr3* expression in WT mice. Mice from three cohorts were randomly assigned to SC or EE. Cohort 1 included 6 mice in EE and 6 mice in SC, cohort 2 included 4 mice in EE and 4 in SC and cohort 3 included 3 mice in EE and 3 mice in SC. Values represent mean ±SEM (t-test, ***p <0,0001).

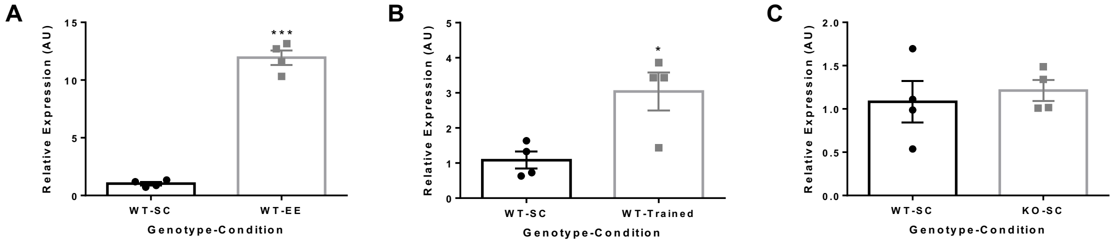

Supplemental Figure 2: Relative *Ryr2* expression in different conditions. **A,** Hippocampal *Ryr2* mRNA quantification for WT mice reared in EE relative to SC housed mice; t-test ***P <0,001 (n = 4). **B**, Hippocampal *Ryr2* mRNA quantification for WT mice trained in the Morris maze relative to SC housed mice that were not trained; t- test *p < 0.05) (n=4). **C**, Expression of *Ryr2* in *Mecp2-null* mice relative to WT mice reared in SC (n = 4).
